# Analyses of m6A regulatory genes and subtype classification in atrial fibrillation

**DOI:** 10.3389/fncel.2023.1073538

**Published:** 2023-06-26

**Authors:** Yingliang Zhao, Yanyun Che, Qiming Liu, Shenghua Zhou, Yichao Xiao

**Affiliations:** ^1^Department of Cardiovascular Medicine, Second Xiangya Hospital of Central South University, Changsha, Hunan, China; ^2^Xiangya School of Medicine, Central South University, Changsha, Hunan, China

**Keywords:** atrial fibrillation, m6A regulatory genes, bioinformatics, predictive model, m6A subtypes

## Abstract

**Objective:**

To explore the role of m6A regulatory genes in atrial fibrillation (AF), we classified atrial fibrillation patients into subtypes by two genotyping methods associated with m6A regulatory genes and explored their clinical significance.

**Methods:**

We downloaded datasets from the Gene Expression Omnibus (GEO) database. The m6A regulatory gene expression levels were extracted. We constructed and compared random forest (RF) and support vector machine (SVM) models. Feature genes were selected to develop a nomogram model with the superior model. We identified m6A subtypes based on significantly differentially expressed m6A regulatory genes and identified m6A gene subtypes based on m6A-related differentially expressed genes (DEGs). Comprehensive evaluation of the two m6A modification patterns was performed.

**Results:**

The data of 107 samples from three datasets, GSE115574, GSE14975 and GSE41177, were acquired from the GEO database for training models, comprising 65 AF samples and 42 sinus rhythm (SR) samples. The data of 26 samples from dataset GSE79768 comprising 14 AF samples and 12 SR samples were acquired from the GEO database for external validation. The expression levels of 23 regulatory genes of m6A were extracted. There were correlations among the m6A readers, erasers, and writers. Five feature m6A regulatory genes, ZC3H13, YTHDF1, HNRNPA2B1, IGFBP2, and IGFBP3, were determined (*p* < 0.05) to establish a nomogram model that can predict the incidence of atrial fibrillation with the RF model. We identified two m6A subtypes based on the five significant m6A regulatory genes (*p* < 0.05). Cluster B had a lower immune infiltration of immature dendritic cells than cluster A (*p* < 0.05). On the basis of six m6A-related DEGs between m6A subtypes (*p* < 0.05), two m6A gene subtypes were identified. Both cluster A and gene cluster A scored higher than the other clusters in terms of m6A score computed by principal component analysis (PCA) algorithms (*p* < 0.05). The m6A subtypes and m6A gene subtypes were highly consistent.

**Conclusion:**

The m6A regulatory genes play non-negligible roles in atrial fibrillation. A nomogram model developed by five feature m6A regulatory genes could be used to predict the incidence of atrial fibrillation. Two m6A modification patterns were identified and evaluated comprehensively, which may provide insights into the classification of atrial fibrillation patients and guide treatment.

## 1. Introduction

Atrial fibrillation (AF) is one of the most prevalent arrhythmias with rapid and disordered atrial contraction in clinical practice, and its main mechanism is atrial remodeling, including electrical and structural remodeling (Wakili et al., 2011). The incidence of atrial fibrillation increases with age and is closely related to other heart diseases, such as myocardial infarction and heart failure, which has become a public health problem that cannot be ignored (Ruddox et al., 2017). However, the mechanism of atrial fibrillation occurrence and development is not fully understood, and effective prevention and treatment methods are still relatively limited. Therefore, it is urgent to clarify the specific molecular mechanism of atrial fibrillation as soon as possible.

The chemical modification of RNA by all living organisms exceeds 160 different types (Boccaletto et al., 2018). Of all RNA modifications, N6-methyladenosine (m6A) is the most extensive internal methylation modification at the N6 position of adenosine (Huang et al., 2020). Many biological processes are regulated by m6A in eukaryotes, and its regulatory genes include writers, readers, and erasers, which influence the occurrence and development of a variety of diseases. Currently, studies on m6A mainly focus on tumors, and it has been confirmed that m6A is closely related to lung cancer, cervical cancer, and other cancers (Wang et al., 2020; Yin et al., 2021). However, in cardiovascular diseases, there are few studies on m6A, which mainly focus on heart failure, hypertension, coronary atherosclerotic heart disease, etc., and there are still no clear results in the related studies on atrial fibrillation (Mo et al., 2019; Berulava et al., 2020; Guo et al., 2020). Therefore, clarification of how m6A regulatory genes are involved in AF may provide new insights into the mechanism, prevention, and treatment of AF.

**FIGURE 1:**
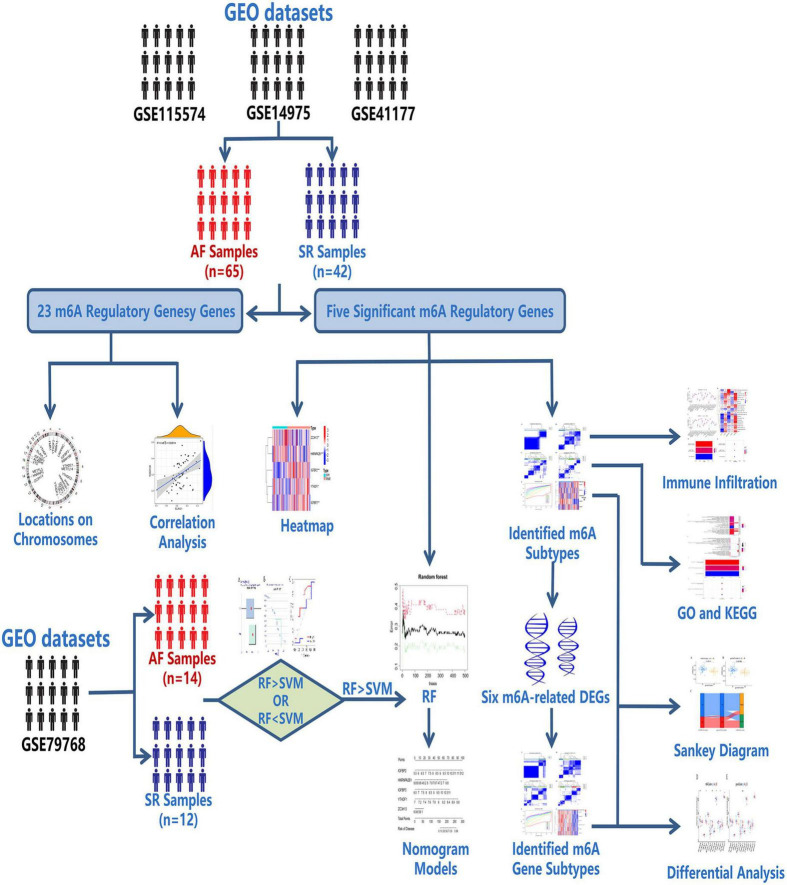
Flow chart of research design and analysis. The data of 65 AF samples and 42 SR samples from three datasets, GSE115574, GSE14975 and GSE41177, were acquired from the GEO database. The expression levels of 23 m6A regulatory genes were extracted. Their locations on chromosomes were located, and correlation analysis was conducted among them. Significant m6A regulatory genes were screened out. The random forest (RF) model was proven superior to the support vector machine (SVM) model. Feature genes in significant m6A regulatory genes were determined by the RF model to establish a predictive nomogram model. The data of 14 AF samples and 12 SR samples from dataset GSE79768 were used for external validation. The m6A subtypes were identified based on the significant m6A regulatory genes, and analysis of immune infiltration was conducted between m6A subtypes. The m6A-related differentially expressed genes (DEGs) were screened between m6A subtypes to conduct Gene Ontology (GO) enrichment analysis and Kyoto Encyclopedia of Genes and Genomes (KEGG) enrichment analysis and genotype the AF samples into m6A gene subtypes. Differential analysis of the m6A score computed by principal component analysis (PCA) algorithms and proven AF-related genes was conducted between the results of the two genotyping methods. The consistency of m6A subtypes, m6A gene subtypes and m6A scores was demonstrated in a Sankey diagram. *p < 0.05, **p < 0.01, and ***p < 0.001.

In the present study, we comprehensively evaluated the significance of the AF subtype results of two genotyping methods based on the GSE115574, GSE14975, and GSE41177 datasets from the Gene Expression Omnibus (GEO) database. We established a nomogram model predicting the incidence of AF based on five m6A regulatory genes (ZC3H13, YTHDF1, HNRNPA2B1, IGFBP2, and IGFBP3). In addition, we revealed that the results of the two genotyping methods were highly consistent in different aspects, which may be meaningful to the classification and treatment of AF. The research flow and rationale are shown in Figure 1.

## 2. Materials and methods

### 2.1. Data acquisition and preprocessing

We searched for expression microarrays that matched terms of atrial fibrillation in the GEO database. The top organisms were filtered by “Homo sapiens,” entry type was filtered by “Series,” and study type was filtered by “Expression profiling by array.” The datasets from the same platform, gathered from clinical studies investigating subjects over 18 years old, employing atrial tissue with experiments (AF) and controls (SR), were included.

R version 4.2.1 was employed for all data processing. All datasets were preprocessed by the R packages “impute” and “limma.” Annotated gene symbols with gene probes. Missing value imputations were performed. Excluded probes without matching gene symbols and average genes with multiple probes. The gene expression quantity with a value greater than 100 was logged, and the gene expression quantity with a value smaller was not processed. Then, datasets were merged and processed to eliminate batch effects and analyzed using the R packages “limma” and “sva.” Finally, each sample was annotated as “treat” or “con” to distinguish the AF samples from the SR samples.

### 2.2. Extraction and differential analysis of m6A regulatory genes

Twenty-three m6A regulatory genes were collected, including seven writers, fourteen readers, and two erasers. The significant m6A regulatory genes with statistically significant expression levels between the AF samples and the SR samples were screened out and visualized (*p* < 0.05). A boxplot was plotted employing the “ggpubr” package, and a heatmap was plotted employing the “pheatmap” package in R software. Perl language was used to make each extracted m6A regulatory gene correspond to its chromosomal location, which was visualized by the R package “RCircos.”

### 2.3. Correlation analysis among m6A readers, writers, and erasers

Spearman analysis was conducted with the R packages “ggplot2,” “ggpubr” and “ggExtra” to explore and visualize the correlations among these m6A regulatory genes. The absolute value of the correlation coefficient was set to no less than 0.4. The *p*-value was set to no less than 0.001.

### 2.4. Establishment of the RF, SVM, and nomogram models

Classifiers based on machine learning, random forest (RF) and support vector machine (SVM), were established to determine feature m6A regulatory genes among significant m6A regulatory genes and predict the incidence of atrial fibrillation. The expression levels of significant m6A regulatory genes in 107 samples from GSE115574, GSE14975 and GSE41177 were used as training data, while 26 samples from GSE79768 were used for external validation. The accuracy of the two models was compared by plotting “boxplots of residual,” “reverse cumulative distribution of residual” and “receiver operating characteristic (ROC)” curves. The RF model was established by the “randomForest” package in R software, and in this model, it was set to 3 and 500 for mtry and ntree, respectively. The importance of significant m6A regulatory genes was assessed according to the selected optimal ntree. We employed the “rms” package in R software to depict a nomogram model that can predict the incidence of atrial fibrillation according to feature m6A regulatory genes. A calibration graph is used to compare the projected value with the actual value. Additionally, a decision curve analysis (DCA) was conducted, and a clinical impact curve was depicted to determine the benefits of the model for patients.

### 2.5. Consensus clustering analysis and analysis of differentially expressed levels of immune cell infiltration

We employed the unsupervised clustering algorithm by the “ConsensusClusterPlus” package in R software. On the basis of the consensus level of significant m6A regulatory genes, we divided the AF samples into diverse subtypes. The most reasonable number of subtypes was decided on the delta area plot and consensus cumulative distribution function (CDF) curves. Additionally, we performed principal component analysis (PCA) to assess the classification. Employing single-sample gene set enrichment analysis (ssGSEA), we computed the AF samples’ immune cell abundance. Furthermore, on the basis of the ssGSEA score, immuno-correlation analysis was performed. Heatmaps and boxplots were generated to display the results.

### 2.6. Identification of m6A gene subtypes based on the DEGs among m6A subtypes

Differentially expressed genes (DEGs) among m6A subtypes were screened out and used to conduct Gene Ontology (GO) enrichment analysis and Kyoto Encyclopedia of Genes and Genomes (KEGG) enrichment analysis. Then, DEGs were used to perform an unsupervised clustering algorithm, dividing AF samples into different m6A gene subtypes. The PCA technique was utilized to compute a m6A score for each AF sample. Difference analysis of the m6A score between the m6A subtypes or m6A gene subtypes was performed. A Sankey diagram was plotted by the R packages “ggalluvial,” “ggplot2” and “dplyr” to demonstrate the consistency among the m6A subtypes, m6A gene subtypes and m6A scores.

### 2.7. Differential analysis of AF-related genes in different subtypes

Some AF-related genes were chosen to perform differential analysis in m6A subtypes and m6a gene subtypes, including SCN5A, KCNH2, TBX3, TBX5, NKX2-5, PITX2, PRRX1, KCNJ5, CASQ2, PKP2, GJA5, KCNJ2, and MYH7 (Roselli et al., 2018). The m6A methylation status of these genes was checked in the m6A-Atlas and directRMDB databases (Tang et al., 2021; Zhang et al., 2023). Heart tissue-specific methylation information was checked in m6A-TShub (Song et al., 2022).

## 3. Results

### 3.1. Data collection

The data of 107 samples from three datasets, GSE115574, GSE14975 and GSE41177, were downloaded from the GEO database for training, including 42 SR samples and 65 AF samples. The data from the three datasets were normalized, merged, and processed by R language to eliminate batch effects. The data from the GSE79768 dataset, which included 14 AF samples and 12 SR samples, were downloaded from the GEO database and normalized for external validation.

### 3.2. Landscape of the 23 m6A regulatory genes in atrial fibrillation

**FIGURE 2:**
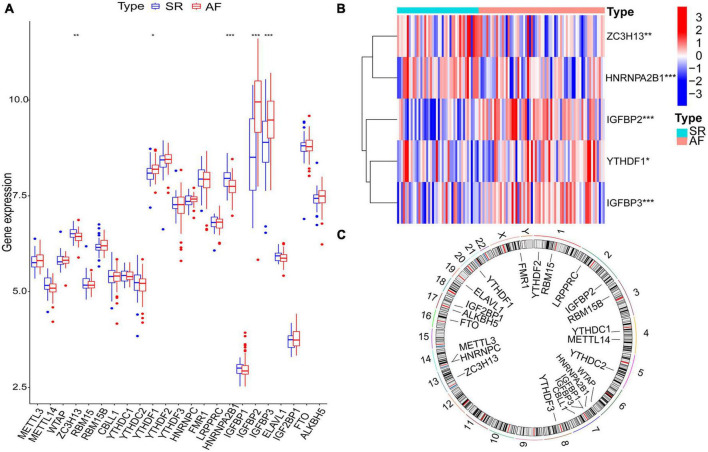
The m6A regulatory genes in AF samples and SR samples. **(A)** The differential expression boxplot of 23 m6A regulatory genes between patients with AF and SR. **(B)** The expression heatmap of five significant m6A regulatory genes in the AF samples and SR samples. **(C)** The chromosomal position of the m6A regulatory genes. *p < 0.05, **p < 0.01, and ***p < 0.001.

Twenty-three m6A regulatory genes were extracted, including seven writers, fourteen readers, and two erasers (Table 1). The differences in the expression levels of 23 m6A regulatory genes between the AF samples and SR samples are presented (Figure 2A). Five genes with statistically significant expression levels (ZC3H13, YTHDF1, HNRNPA2B1, IGFBP2, and IGFBP3) were screened out (*p* < 0.05). IGFBP2, YTHDF1, and IGFBP3 were upregulated in the AF samples and downregulated in the SR samples. In contrast, ZC3H13 and HNRNPA2B1 were downregulated in the AF samples and upregulated in the SR samples. The heatmap of the five significant genes was plotted (Figure 2B). The locations of the m6A regulatory genes on chromosomes are presented in Figure 2C.

**TABLE 1:** Twenty-three m6A regulatory genes extracted in this study.

Type	m6A regulatory genes
m6A writer	METTL3
	METTL14
	WTAP
	ZC3H13
	RBM15
	RBM15B
	CBLL1
m6A reader	YTHDC1
	YTHDC2
	YTHDF1
	YTHDF2
	YTHDF3
	HNRNPC
	FMR1
	LRPPRC
	HNRNPA2B1
	IGFBP1
	IGFBP2
	IGFBP3
	ELAVL1
	IGF2BP1
m6A eraser	FTO
	ALKBH5

Through atrial tissue gene analysis between patients with atrial fibrillation (AF) and sinus rhythm (SR), we extracted 23 m6A regulatory genes from the datasets, including seven writers, fourteen readers, and two erasers.

### 3.3. Correlation among m6A readers, writers, and erasers in atrial fibrillation

**FIGURE 3:**
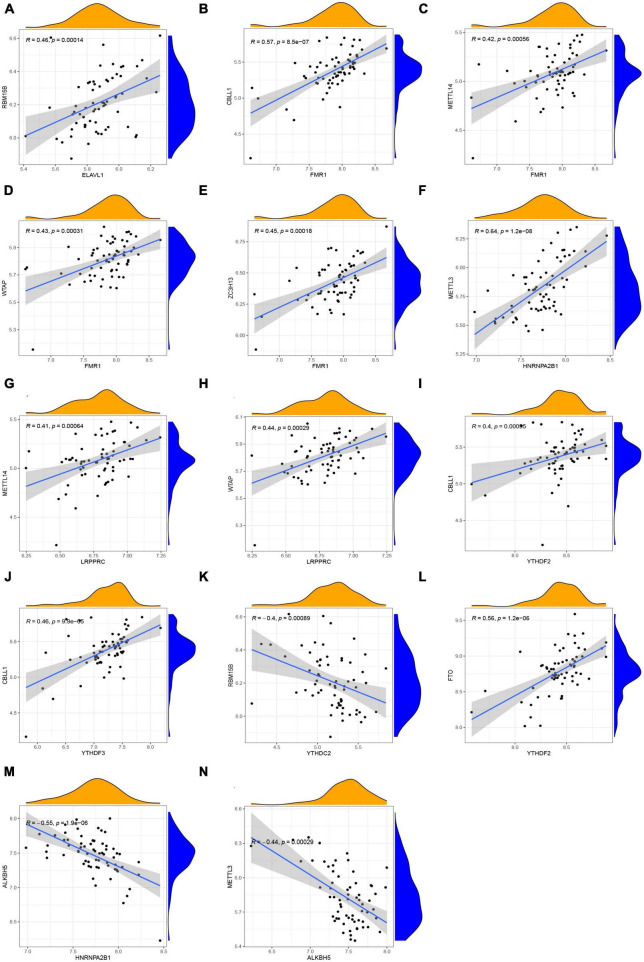
Correlation among m6A readers, writers, and erasers in AF. **(A–N)** Reader genes: ELAVL1, FMR1, HNRNPA2B1, LRPPRC, YTHDF2, YTHDF3, and YTHDC2; Writer genes: RBM15B, CBLL1, METTL14, WTAP, ZC3H13, and METTL3; Erasers genes: FTO and ALKBH5 (| R| > 0.4 and *p* < 0.001). R, correlation coefficient.

How m6A readers, writers, and erasers correlated with each other was examined by linear regression analyses. In general, it showed high positive correlations between readers’ and writers’ expression levels (Figures 3A–J), while readers YTHDC2 displayed negative correlations with writer RBM15B (Figure 3K). The most significant correlation was between reader HNRNPA2B1 and writer METTL3, with a correlation coefficient of 0.64 and a *p*-value of 1.2e-08 (Figure 3F). Positive correlations were also found between the reader YTHDF2 and eraser FTO (Figure 3L). Atrial fibrillation patients with a high level of the eraser ALKBH5 showed low expression levels of the reader HNRNPA2B1 and writer METTL3 (Figures 3M, N). Further details are provided in Supplementary File 1.

### 3.4. Establishment of the RF, SVM, and nomogram models

**FIGURE 4:**
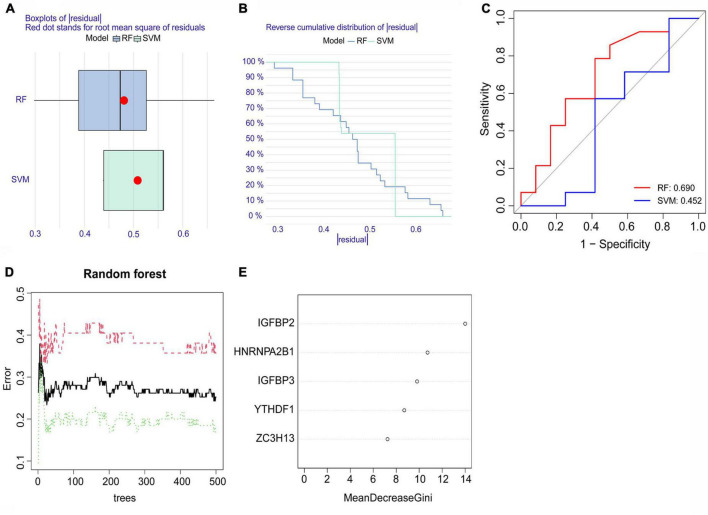
The random forest (RF) model and support vector machine (SVM) model. **(A)** A lower residual value was seen in the boxplots of residuals for the RF model. **(B)** The residual distribution of the RF model and SVM model was demonstrated with the reverse cumulative distribution of residuals. **(C)** The ROC curves showed the RF model with a 0.690 predictive value and the SVM model with a 0.452 predictive value. **(D)** The red curve represents the error levels of AF groups, the green curve represents SR groups, and the black curve represents overall samples. **(E)** The importance scores of the five significant m6A regulatory genes.

Both the RF model and SVM model were developed using training data to determine feature m6A regulatory genes within the five significant m6A regulatory genes to characterize disease and forecast the incidence of atrial fibrillation. Using the data from dataset GSE79768 for external validation, the results of the boxplots of residual, reverse cumulative distribution of residual, and ROC curves (Figures 4A–C) revealed that the RF model predicts more accurately, indicating that this model was superior to the SVM model. The RF model showed a lower residual value and a larger area under the ROC curve than the SVM model. This can be attributed to the RF model’s ability to handle complex interactions and non-linear relationships among variables, as well as reducing overfitting and bias by averaging the outcomes of multiple decision trees. As a result, the RF model was chosen (Figure 4D). We checked the five significant m6A regulatory genes after they were ranked according to their importance (Figure 4E).

**FIGURE 5:**
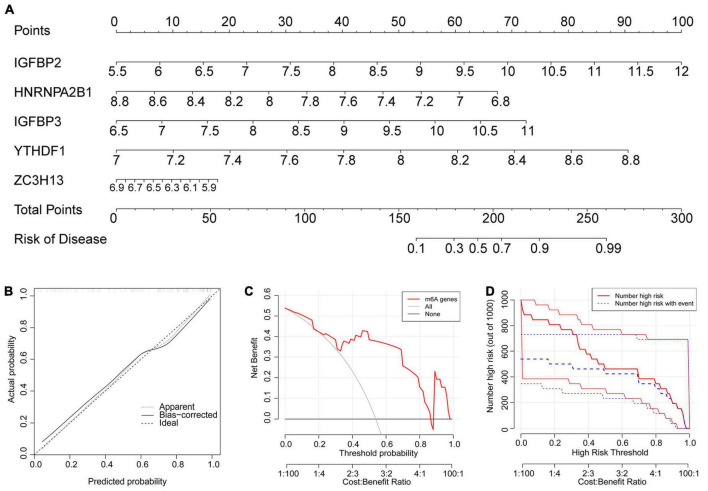
The nomogram model was established. **(A)** The nomogram model was established according to the five feature m6A regulatory genes. The incidence was predicted according to total points. A total of 160 points indicated an incidence of 10%, while 225 points indicated an incidence of 90%. **(B)** The solid and dotted lines are close in the calibration curves, indicating the strong predictive power of the nomogram model. **(C)** The red line representing the m6A genes in the decision curve deviating from the gray and black lines also proved the feasibility of the model. **(D)** The red lines represent patients with a high risk of AF, and blue lines represent patients with AF. The clinical impact curve verified that the model can benefit patients clinically.

Because their importance scores were all greater than 2, they were all qualified for establishing the nomogram model (Figure 5A). In the nomogram model, each gene can be scored individually. The total score that can predict the incidence of atrial fibrillation is computed by adding up the scores. The data from dataset GSE79768 were used for external validation again. The solid and dotted lines were close in the calibration curves (Figure 5B). The red line representing the m6A genes in the decision curve deviated from the gray and black lines (Figure 5C). Both the above graphs and the clinical impact curve (Figure 5D) indicated that the model demonstrates promising potential for prognosis in atrial fibrillation patients. However, we acknowledge the need for further validation with larger and more diverse datasets to confirm its prognostic capability.

### 3.5. Two m6A subtypes identified by significant m6A regulatory genes

**FIGURE 6:**
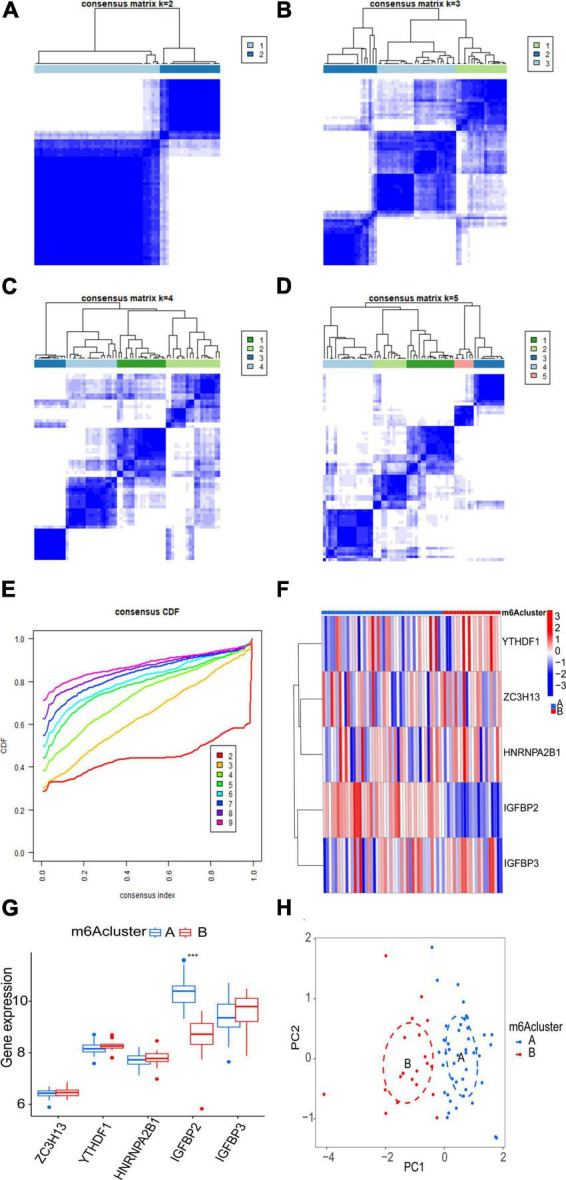
Clustering analysis of five significant m6A regulatory genes associated with AF. **(A–D)** Consensus matrices display the clustering of the five significant m6A regulatory genes for k values ranging from 2 to 5. **(E)** The k value of 2 exhibits the smallest descending grade in the consensus cumulative distribution function (CDF) curve, indicating optimal clustering. **(F,G)** Expression heatmap and boxplot demonstrate the differential expression of the five significant m6A regulatory genes between cluster A and cluster B. **(H)** Principal component analysis (PCA) illustrates the distinct expression patterns of m6A subtypes. Statistical significance is denoted as follows: ***p < 0.001.

The consensus clustering algorithm in the R package “ConsensusClusterPlus” was adopted to identify m6A subtypes according to the five significant m6A regulatory genes. The results showed that the AF samples were divided into two m6A subtypes (Figures 6A–E). Eighteen samples belonged to Cluster A, while 47 samples belonged to Cluster B (Supplementary File 2). To demonstrate the differential expression of the five significant m6A regulatory genes between the two clusters, a heatmap and a boxplot were graphed (Figures 6F, G). There was evidently a greater level of IGFBP2 expression in cluster A (*p* < 0.001).

**FIGURE 7:**
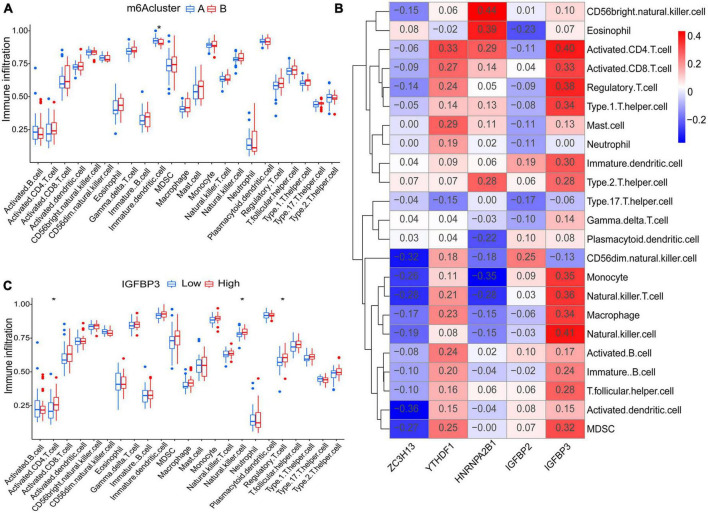
Analyses based on ssGSEA results. **(A)** Differences in immune cell infiltration between cluster A and cluster B. **(B)** Immuno-correlation analysis was performed between immune cells and five significant m6A regulatory genes. IGFBP3 was the most strongly associated with immune cells. **(C)** Differential immune cell infiltration between groups with lower and higher IGFBP3 expression. *p < 0.05.

Principal component analysis of the expression of the five m6A regulatory genes indicated that the two m6A subtypes could be distinguished by the five genes (Figure 6H). Employing ssGSEA, we computed AF sample immune cell abundance. In this study, we examined the differences in immune cell infiltration between clusters A and B (Figure 7A). Compared to cluster B, cluster A displayed a higher number of infiltrated immature dendritic cells (*p* < 0.05). In addition, an examination of the relationship between five significant m6A regulatory genes and immune cells was conducted (Figure 7B). The outcome showed that immune cells were most strongly associated with IGFBP3. We performed correlational analysis between IGFBP3 and immune cells (Figure 7C). There was an increase in immune cell infiltration of regulatory T cells (*p* < 0.05), natural killer cells (*p* < 0.05), and activated CD4 T cells (*p* < 0.05) in samples with higher IGFBP3 expression.

### 3.6. Identification of two m6A gene subtypes and consistency check between two genotyping methods

**FIGURE 8:**
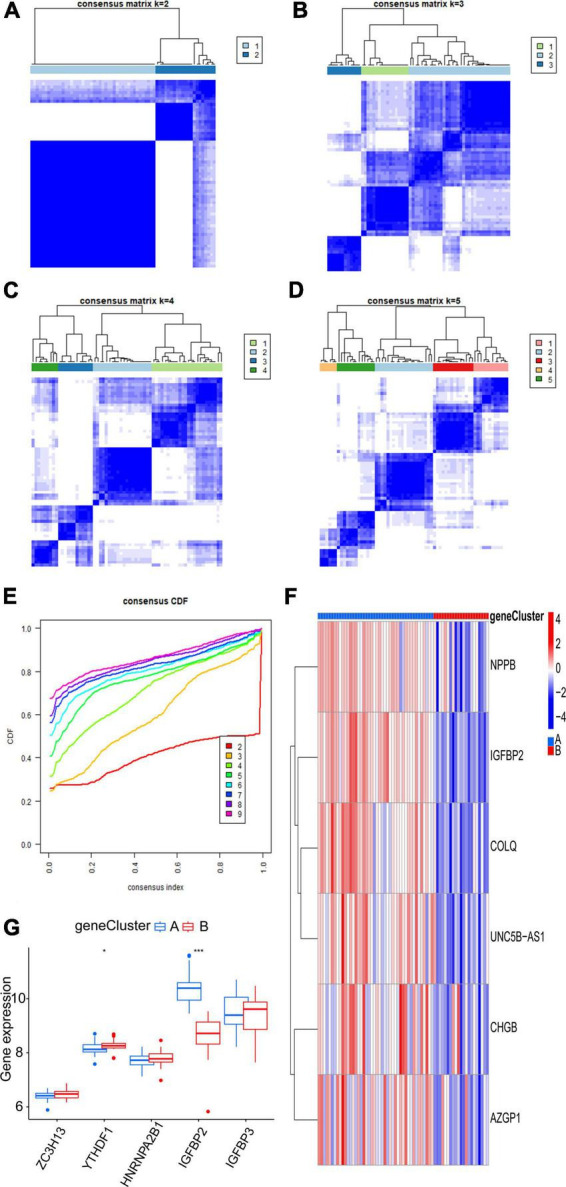
Consensus clustering of the six m6A-related DEGs. **(A–D)** Consensus matrices of the six m6A-related DEGs (*k* = 2–5). **(E)** The k value of 2 had the smallest descending grade in the consensus CDF curve. **(F)** Expression heatmap of six feature m6A-related DEGs between gene cluster A and gene cluster B. **(G)** Expression boxplot of the five significant m6A regulatory genes between gene cluster A and gene cluster B. *p < 0.05, ***p < 0.001.

**FIGURE 9:**
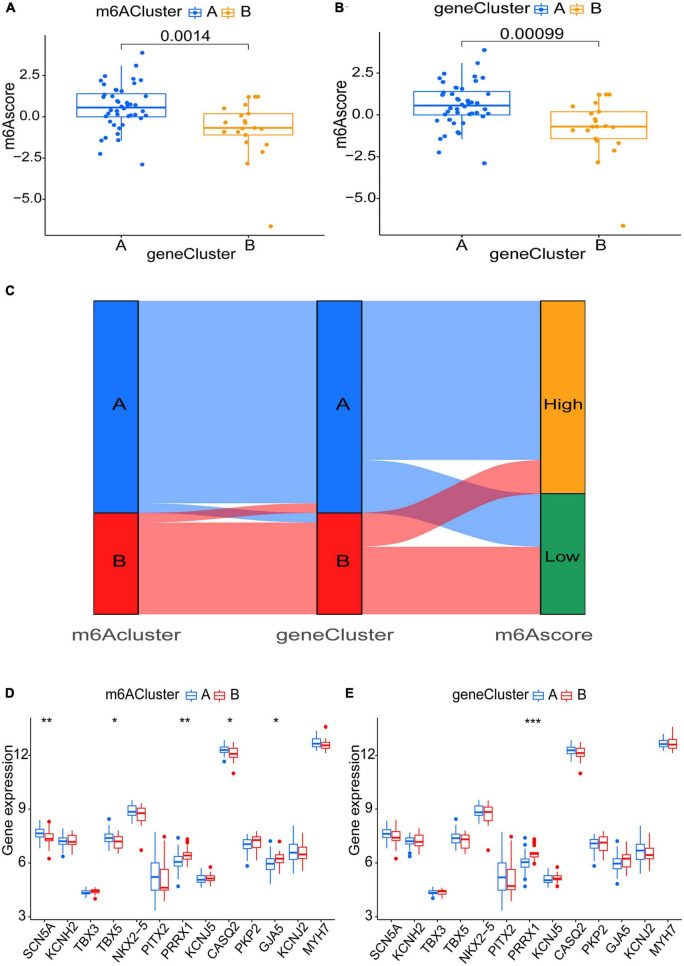
Role of m6A subtypes and m6A gene subtypes in distinguishing AF. **(A,B)** m6A score differences between m6A subtypes or m6A gene subtypes (*P* < 0.05). **(C)** Sankey diagram demonstrating the consistency among m6A subtypes, m6A gene subtypes, and m6A scores. **(D,E)** Differential expression levels of SCN5A, KCNH2, TBX3, TBX5, NKX2-5, PITX2, PRRX1, KCNJ5, CASQ2, PKP2, GJA5, KCNJ2, and MYH7 between m6A subtypes or m6A gene subtypes. *p < 0.05, **p < 0.01, and ***p < 0.001.

Six m6A-related DEGs with statistically significant expression levels were identified between the two m6A subtypes and were included in the KEGG and GO functional enrichment analyses. The top 10 biological processes, cellular components, and molecular functions are shown in Supplementary Figure 1B. The top 10 KEGG pathways are shown in Supplementary Figure 1D. These DEGs were mainly enriched in the following pathways: vascular smooth muscle contraction, cGMP-PKG signaling pathway, and thermogenesis. The consensus clustering algorithm was employed again. On the basis of six m6A-related DEGs between two m6A subtypes, two m6A gene subtypes were identified (Figures 8A–E), which was similar to the genotyping results of m6A subtypes (Supplementary File 3). How the six m6A-related DEGs were expressed in m6A gene subtypes is displayed in the heatmap (Figure 8F). Similar to the m6A subtypes, IGFBP2 expression was evidently higher in gene cluster A (*p* < 0.001) (Figure 8G). To exactly quantify the m6A subtypes, PCA was adopted to compute the m6A score. A differential analysis of the m6A score was performed between m6A subtypes or m6A gene subtypes. The outcome indicated that cluster A or gene cluster A scored statistically higher than the other clusters (*p* < 0.05) (Figures 9A, B). In the Sankey diagram, we observed high consistency among the m6A subtypes, m6A gene subtypes, and m6A scores (Figure 9C).

### 3.7. Role of m6A genotyping in classifying atrial fibrillation

To uncover the link between atrial fibrillation and the results of the two genotyping methods, we examined the correlation between the two genotyping results and SCN5A, KCNH2, TBX3, TBX5, NKX2-5, PITX2, PRRX1, KCNJ5, CASQ2, PKP2, GJA5, KCNJ2, and MYH7. These genes have been verified to be involved in AF (Roselli et al., 2018). All these genes are present in m6A-Atlas, while KCNH2, TBX3, NKX2-5, PITX2, PRRX1, KCNJ5, PKP2, and KCNJ2 are present in directRMDB due to the two databases using different techniques (Tang et al., 2021; Zhang et al., 2023). Unfortunately, these genes are not specifically methylated in heart tissues according to m6A-TShub (Song et al., 2022). The outcome demonstrated that both cluster A (*p* < 0.01) and gene cluster A (*p* < 0.001) had lower PRRX1 expression levels, which indicated that cluster A and gene cluster A may have action potential shortening (Tucker et al., 2017; Figures 9D, E).

## 4. Discussion

We screened five significant m6A regulatory genes from a total of 23 extracted m6A regulatory genes. With the RF model, we established a nomogram model to predict the incidence of AF based on the five feature genes. Then, we genotyped the AF samples according to five significant m6A regulatory genes and six m6A-related DEGs. Furthermore, we performed diverse analyses, including m6A regulatory gene expression, immune infiltration, PCA, and AF-related gene expression, of the genotyping results, discovering that the subtype results of the two genotyping methods were consistent.

Atrial fibrillation is the most common serious arrhythmia in clinical practice. Despite extensive research on AF pathogenesis and treatment, therapeutic outcomes remain suboptimal, and AF continues to be a significant contributor to mortality and healthcare expenditure (Zimetbaum, 2017; Hu et al., 2019; Qin et al., 2019). Recent studies have indicated that m6A, as an essential regulator, is involved in cancer development (Zhang et al., 2017; Cai et al., 2018). However, there are still limited studies on the mechanism of m6A regulatory genes in atrial fibrillation. Thus, investigating how m6A regulatory genes are involved in AF, constructing a nomogram model to predict the incidence, classifying AF patients according to m6A regulatory genes, and clarifying genotyping significance will be helpful for AF treatment and prevention.

In this study, we focused on the relevance of our findings to the regulatory mechanisms leading to AF and their potential clinical applicability. We provided concise information regarding the potential biological and clinical translation of our findings. In our study, we found that IGFBP2, IGFBP3, and YTHDF1 were upregulated in AF samples, highlighting their potential biological significance in AF development and progression. IGFBP-2 is crucial for VEGF expression and angiogenesis (Azar et al., 2014). VEGF-D concentrations have been associated with AF (Berntsson et al., 2019), suggesting IGFBP-2’s role in AF through VEGF-D regulation. IGFBP2 promotes ERK phosphorylation in an integrin-dependent manner (Han et al., 2014), which could link it to atrial fibrosis and AF pathogenesis via cellular signaling pathways (Goette et al., 2000). IGFBP2 activates integrin β1 and downstream pathways, requiring ILK for cell motility induction and NF-κB activation (Holmes et al., 2012). NF-κB is involved in inflammatory cytokines, thrombogenesis, and fibrosis gene expression (Harada et al., 2015), suggesting that the IGFBP2/integrin/ILK/NF-κB network may contribute to AF development and progression. IGFBP2 suppresses PTEN and promotes vascular smooth muscle cell growth through enhanced PTEN tyrosine phosphorylation via dimerization with RPTPβ (Shen et al., 2012). Given the role of the PTEN/AKT/mTOR pathway in cardiac hypertrophy and fibrosis (Sun et al., 2021), IGFBP2’s modulatory effect on PTEN may influence AF development. m6A modifications may control IGFBP3 expression during cardiac fibrosis development (Ding et al., 2023). Silencing METTL3 led to downregulation of IGFBP3 expression and inhibition of cardiac fibroblast activation and fibrosis (Ding et al., 2023). Low IGF1/IGFBP3 ratios are associated with higher AF prevalence (Busch et al., 2019). ALKBH5-mediated m6A modification increased YTHDF1 expression, promoting the translation of Yes-associated protein (YAP), a core regulator of heart regeneration (Han et al., 2021). This process may be related to the lack of cardiac repair in AF.

At present, predictors of risk models for predicting AF incidence include classical cardiovascular risk factors, biomarkers, genetic variants, and imaging methods (Yuasa and Imoto, 2016; Borschel et al., 2021; Yang et al., 2022). Several risk models, such as CHARGE-AF and HMS, have been assessed for their accuracy in identifying high-risk individuals (Poorthuis et al., 2021). Genetic risk prediction models have also been developed, with some showing improved AF prediction as the number of SNPs increases (Lubitz et al., 2017; Borschel et al., 2021). However, no study has used m6A regulatory gene expression to establish a predictive model. Our nomogram model provides a convenient tool for predicting AF incidence at the gene expression level but needs validation with larger samples.

Inflammation is one of the risk factors for atrial fibrillation. Inflammation and the immune response caused by it participate in the occurrence and development of AF (Aviles et al., 2003; Hu et al., 2015). A recent review also proposed the concept of immune remodeling in AF, highlighting significant changes in the immune system during AF and its interactions with the cellular and environmental factors involved in AF initiation and maintenance (Yao et al., 2022). Studies suggest that dendritic cells and regulatory T cells may be related to AF pathogenesis (Li et al., 2021; Xiao et al., 2021; Liu et al., 2022; Xie et al., 2022). The PD-1/PD-L1 pathway plays a key role in AF immunomodulation by regulating T-cell activation and promoting inflammatory factor secretion (Liu et al., 2015; Chang et al., 2022). IL-6-miR-210 can inhibit regulatory T-cell function by targeting Foxp3, promoting atrial fibrosis and leading to AF development (Wang et al., 2021). Our study found differences in immune cell infiltration, including immature dendritic cells, regulatory T cells, natural killer cells, and activated CD4 T cells, between m6A subtypes and AF samples divided by IGFBP3 expression. These findings imply that m6A may be involved in AF development by regulating immune infiltration.

Atrial fibrillation is thought to depend on abnormal pulse formation, conduction, and the propensity to reenter the ostium of the pulmonary veins. Most AF-causing foci are located near the pulmonary veins’ ostium, where myocardial cells and vascular smooth muscle cells interlace (Wijffels et al., 1995; Haissaguerre et al., 1998; Carballo et al., 2018). Gap junction proteins in cardiomyocytes are regulated by the phenotypic transition of pulmonary vein vascular smooth muscle cells, leading to heterolytic junctions and AF occurrence (Zhou et al., 2020). KEGG analysis revealed that the vascular smooth muscle contraction pathway was significantly enriched, suggesting that the m6A gene may be involved in regulating vascular smooth muscle cells on cardiomyocytes, leading to AF. This finding aligns with previous studies and adds new hints for understanding the mechanism of AF and guiding its treatment.

We analyzed 13 genes related to AF between m6A subtypes and m6A gene subtypes and found that PRRX1 was significantly different between the two genotyping methods. Reduced PRRX1 expression leads to shortened action potentials in cardiomyocytes and may promote AF (Tucker et al., 2017). Another study with over half a million subjects showed that reduced PRRX1 expression was associated with AF (Roselli et al., 2018). The finding that proven AF genes were expressed differently in our classified subtypes indicates that our results are consistent with previous studies and that the classification is meaningful.

Overall, the highlights of our study are establishing a predictive model and proposing new genotyping methods based on m6A regulatory genes, which may improve the prediction of AF clinically and guide the molecular mechanism study of AF. However, there are still some limitations in this research. First, due to the lack of large datasets that meet the conditions in the geo database, we combined three chips from the same platform GP570, which resulted in the batch effect. In our study, we focused on the distinction between AF and control patients, but we acknowledge the importance of differentiating between the clinical subtypes of AF, such as paroxysmal, persistent, and permanent AF. However, due to the limited availability of suitable GEO datasets, we were unable to conduct a comparative study of these subtypes. The challenges in obtaining data on paroxysmal AF and the predominance of persistent and permanent AF cases in existing datasets further complicated the analysis. We hope that there will be enough datasets in the GEO database or collected by us to improve our study. Second, the number of DEGs between m6A subtypes was so small that we could not perform effective GO enrichment analysis and KEGG analysis (Supplementary Figure 1). The specific reasons also need further research. Third, the genes that we examined for correlation with the two genotyping results are not heart tissue-specific methylated according to m6A-TShub (Song et al., 2022). Most importantly, additional experiments should be carried out to elucidate the molecular mechanisms contributing to AF. We would like to solve these limits in the future.

## 5. Conclusion

In conclusion, the diversity of m6A regulatory gene expression patterns has a significant influence on the heterogeneity of atrial fibrillation. The predictive model we established may optimize the prediction of atrial fibrillation. Comprehensive analysis of the two subtypes may contribute to discovering the molecular mechanism of AF and guiding treatment based on individual genotyping.

## Data availability statement

Publicly available datasets were analyzed in this study. This data can be found here: https://www.ncbi.nlm.nih.gov/, GSE115574, GSE14975, GSE41177, and GSE79768.

## Ethics statement

The studies involving human participants were reviewed and approved by the Ethics Committee of Second Xiangya Hospital. It is noted that informed consent was obtained from the patients and individuals involved in the original studies, whose data is publicly available.

## Author contributions

YX designed the whole study. YZ carried out the statistical analysis. YZ and YC completed the original draft of the manuscript. QL, SZ, and YX revised the manuscript. All authors approved the final manuscript.

## Supplementary material

Supplementary File 1 Expression of 23 m6A regulatory genes.

Supplementary File 2 Genotyping results of m6A subtypes.

Supplementary File 3 Genotyping results of m6A gene subtypes.

Supplementary Figure 1 Gene Ontology (GO) enrichment and Kyoto Encyclopedia of Genes and Genomes (KEGG) analyses.
