# Supplementary figures and images for: Analyses of m6A regulatory genes and subtype classification in atrial fibrillation
Source: Front Cell Neurosci. 2023 Jun 26;17:1073538. doi: 10.3389/fncel.2023.1073538 (PMC10330950; doi:10.3389/fncel.2023.1073538)

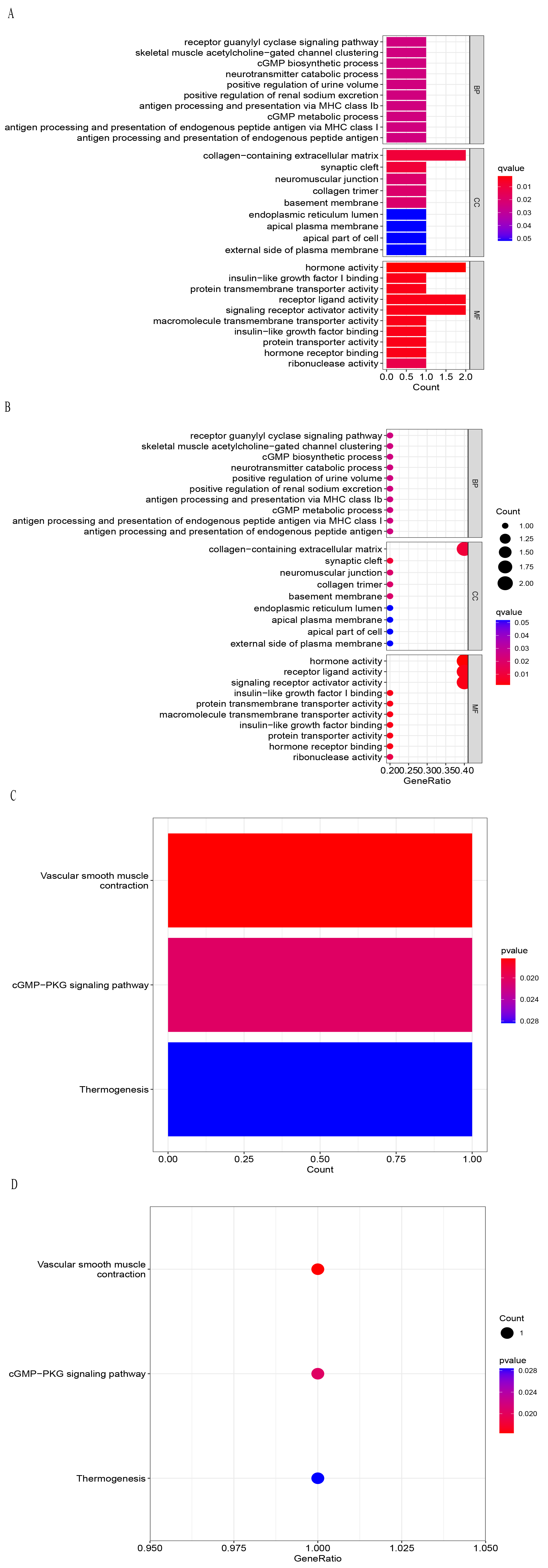

Supplement: Supplementary Figure 1 — Gene Ontology (GO) enrichment and Kyoto Encyclopedia of Genes and Genomes (KEGG) analyses. [file Image_1.TIF]
